# Lessons Learned From Producing Guidance Articles and Rapid Massive Open Online Courses During the Covid-19 Pandemic in Primary Care

**DOI:** 10.1177/2150132720963624

**Published:** 2020-10-01

**Authors:** Mohammad S. Razai, Hadyn K. N. Kankam, George J. M. Hourston, Szymon Hoppe, Pippa Oakeshott

**Affiliations:** 1 St George’s University of London, London, UK; 2 St George’s University Hospital NHS Foundation Trust, London, UK; 3 Addenbrooke’s Hospital, Cambridge, UK; 4 Medical University of Gdansk, Gdansk, Poland; 5 St George’s University of London, London, UK

Baker and colleagues have produced very useful, practical, evidence-based guidelines for healthcare workers about personal protection against SARS-CoV-2 infection.^1^ Throughout the pandemic, there has been a need for such up to date, reliable guidance in primary care that can be delivered virtually. This is crucial for safe and effective management of a novel and rapidly evolving situation.

Educational guidance documents such as those written by Baker et al^1^ might have even more impact if disseminated through innovative technologies such as free, massive open online courses (MOOCs).^2^ These courses benefit from a wide reach and flexibility, which is crucial while the evidence on covid-19 is continually changing. They have been taken up by universities worldwide,^2^ (including St George’s University of London) as a means of widening access, encouraging unlimited learner participation and revolutionizing the field of healthcare training. Peer-reviewed articles produced at speed in high-impact journals provide the evidence-base for the teaching content of these online courses. We would like to share our experience of producing guidance articles and rapid MOOCs for primary care education on covid-19.

In spring 2020, we wrote 2 guides for primary care^3,4^ using expertise from general practice, public health, and infectious diseases. Both guides were published via fast-track pathways: “Covid-19 a guide for UK GPs”^3^ and “Mitigating the psychological impact of social isolation during the covid-19 pandemic.”^4^ We then developed a rapid 3-week MOOC on the free FutureLearn platform (The Open University and SEEK Ltd, London, UK) within a week of publication to support primary care staff.^5^

As the pandemic evolved, mental and psychological effects due to social isolation also became a matter of public health concern, which the second guidance sought to address.^4^ A second 2-week MOOC was developed for African primary care units in partnership with Ilara Health (Nairobi, Kenya).^6^ These courses aimed to provide frontline primary care staff working in a very different environment from the UK with the support they need to manage patients while protecting themselves and their communities.

We were keen to make the courses practical and concise. Each week we presented a variety of learning resources including recently published articles, infographics, audio interviews, quizzes, and frequently asked questions. At the end of each week, there was an opportunity to participate in discussion with fellow learners from around the world. Both MOOCs had over 30 000 learners from 180 countries in their first runs. Overall participants rated the UK and Africa courses 4.7/5 and 4.8/5, respectively. Comments from primary care included: “The course provided clear, logical information presented at a good pace. The resources are excellent. The opportunity to learn from others’ experiences and views was enlightening and valuable.” “I have learnt a lot and this course gives very practical resources and guidance.” “As a community Palliative Care Nurse I found your advice on telephone/video assessments and assessing the breathless patient via a telephone particularly helpful.” “This has been a useful and informative course, the resources for asthma and COPD care are excellent.” “Very practical, and the links and resources are excellent. Also terrific to hear practitioner perspectives from around the world!” Learning points were presented in an international FutureLearn festival as well as in regional and local virtual forums.

The covid-19 pandemic created a unique situation with the challenges of a new disease with little evidence on epidemiology and appropriate management. However, lessons learned from developing educational technologies and guidance articles can be applied in a variety of settings in primary care education. We learned that the MOOCs afforded the flexibility to change the content, update the learning steps and adapt the material based on both the evolution of the pandemic, and learners’ needs and feedback in real-time. We found the MOOCs highly suitable for quick dissemination of knowledge within and beyond the UK. The engagement from learners in virtual discussion groups was particularly useful and was frequently highlighted by participants as an effective way to share tips from around the world.

Another advantage of these courses was the removal of access barriers and the fact that the course was offered free of charge. Provided they had internet, learners could access the information from anywhere in the world. Leading experts in primary care education as well some NHS organizations endorsed the UK MOOC.^5^ The main limitations are that although we provided quizzes at the end of each week, there was no mechanism to assess participants’ learning, how many completed the course and what practical impact the courses had on knowledge and practice. However, this is not different to some continuous professional development methods that are currently widely used.

Guidelines like those written by Baker and colleagues are important in ensuring safe delivery of care. Additionally, free MOOCs developed by experts provide accessible and interactive learning communities that can augment and, in some cases, replace the traditional methods of primary care education. This technological approach to primary care education is particularly important during the current pandemic where face to face interaction is limited by physical distancing measures.
